# Pan-media liquid metal 3D printing

**DOI:** 10.1016/j.xinn.2025.100813

**Published:** 2025-01-27

**Authors:** Xiaohui Shan, Weichen Feng, Minghui Guo, Xuelin Wang, Jing Liu

**Affiliations:** 1 School of Biomedical Engineering, Tsinghua University, Beijing 100084, China; 2 Key Laboratory of Cryogenic Science and Technology, Technical Institute of Physics and Chemistry, Chinese Academy of Sciences, Beijing 100190, China; 3 Beijing Advanced Innovation Center for Biomedical Engineering, Beijing Advanced Innovation Center for Big Data-Based Precision Medicine, School of Engineering Medicine, Beihang University, Beijing 100191, China

## Introduction

The manufacturing industry has evolved from traditional forging and cutting to three-dimensional (3D) printing, a revolutionary technology that expands human imagination by creating everything of complex shapes and intricate structures, making it a cornerstone of smart manufacturing.^1^ However, conventional fabrications may encounter bottlenecks that seriously restrict their printing across different inks, ambient, and post-processing. Adapting existing 3D printing methods to produce target functional components on diverse working media, therefore, requires significant adjustments. As highly adaptable 3D printing inks, liquid metals (LMs) open large spaces to address such challenges due to their versatile capabilities, such as fluidities, conductivities, easy solid-liquid transitions, and more. Although continuous efforts are being increasingly made in LM printing in different media,^2^ there is still no generalized methodology or concept proposed to unite all LM 3D printing techniques, inks, and media together. To achieve this intriguing target, here we present a unified 3D printing concept, termed *pan-media liquid metal 3D printing,* to manufacture customized end-user devices as desired. Pan-media 3D printing means that it is able to administrate printing with any ink and any working ambient, from gases and liquids to soft matters, bio-tissues, and rigid media, transcending the boundaries of traditional printing environments. Particularly, with intentionally introduced physical or chemical processing between LM inks and ambient, pan-media LM 3D printing could achieve a much wider variety of 3D object and targeted functions over existing approaches. By synthesizing the pan-media theory of LM 3D printing, we prospect a pan-media manufacturing center equipped with functional ink storage, media library, printing head system, and control units, all integrated to fully address the desired printing tasks.

## Printing inks

**Figure 1:**
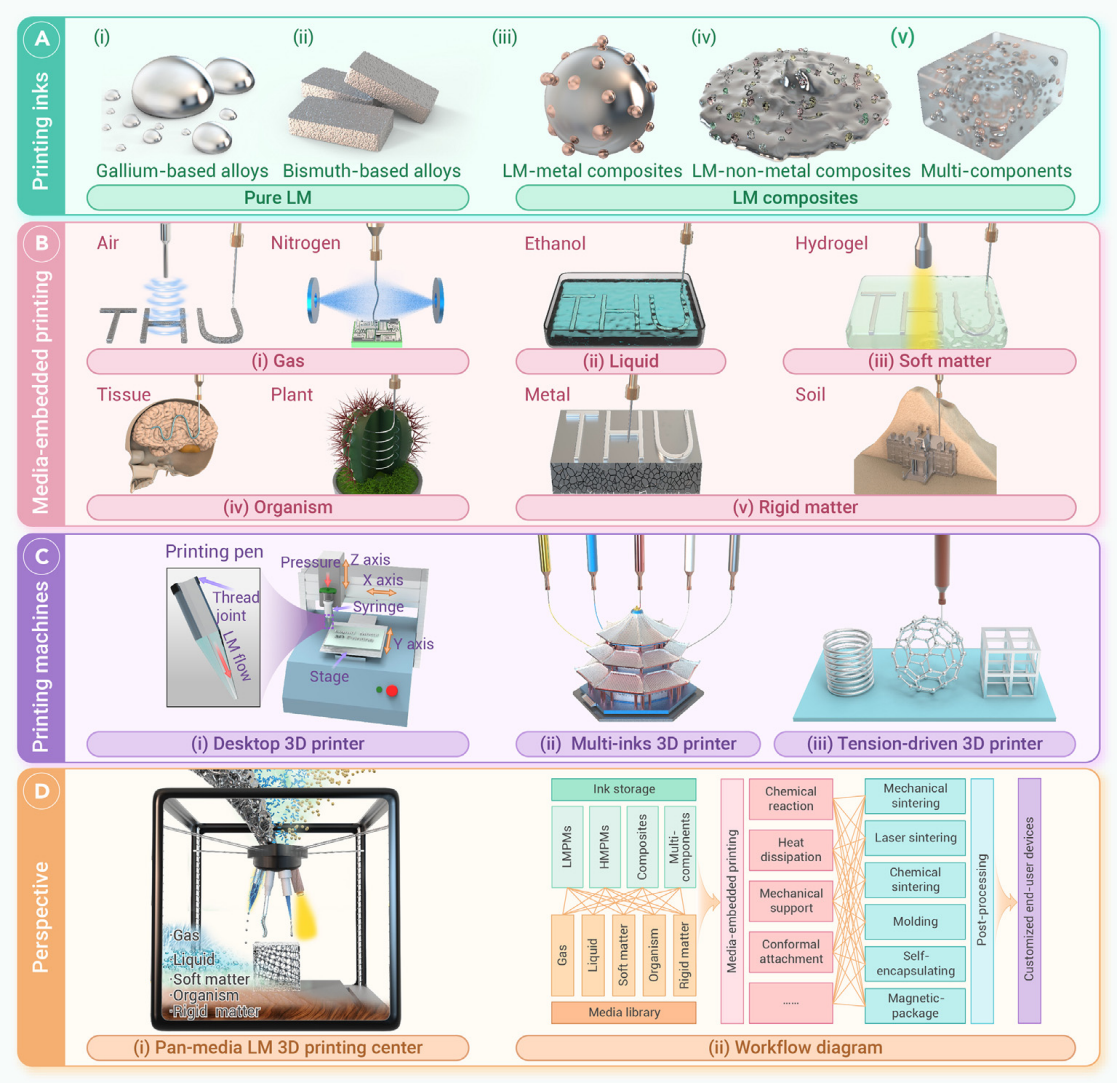
Pan-media liquid metal 3D printing (A) Typical LM printing inks in 3D printing, including pure LM (i, gallium-based alloys and ii, bismuth-based alloys) and LM composites (iii, LM-metal composites; iv, LM-non-metal composites; and v, multi-components). (B) Media-embedded printing methods and media including (i) gas media, (ii) liquid media, (iii) soft matter media, (iv) organism media, and (v) rigid matter media. (C) Classical LM 3D printing machines: (i) desktop 3D printer, modified from Zheng et al.,^4^ (ii) multi-ink 3D printer, and (iii) tension-driven 3D printer, modified from Ling et al.^5^ (D) Perspective of a pan-media LM 3D printing center: (i) profile of pan-media LM 3D printer and (ii) workflow diagram of the designed 3D printing center.

The adoption of the pan-media 3D printing theory significantly broadens the range of printable inks beyond low-melting-point metals (LMPMs), extending to high-melting-point metals (HMPMs), non-metals, and multi-components.^3^ LMPMs, particularly pure LMs (gallium-based alloys [Figure 1Ai] or bismuth-based alloys [Figure 1Aii]), have demonstrated unique advantages in 3D printing thanks to their rapid cooling solidification and layer-by-layer stacking, thus facilitating the manufacture of complex 3D structures. For HMPMs (e.g., Ti, Al, or steel), manufacturing typically requires an energy source to melt the metal, necessitating extensive post-processing and a highly controlled printing environment. Meanwhile, researchers fabricate a series of functional composites by mixing metal particles (e.g., Fe, or Cu) or non-metal particles (e.g., graphene or carbon nanotubes) to enrich their controllable rheological property and structural stability (Figures 1Aiii and 1Aiv). Furthermore, a series of functional multi-components can be designed by mixing metals or non-metals and even polymers with LM inks to facilitate resolution improvements and enhance shape fidelity (Figure 1Av). Overall, the scope of printing inks can be expanded from LMPMs to HMPMs, composites, and multi-components with their unique fluid and phase-changing properties during printing, which affect physical and chemical behaviors across different media, thereby influencing 3D object formation.

## Media-embedded printing

Unlike conventional 3D printing processes confined to specific media, LMs enable versatile media 3D printing across a vast array of environments ranging from gaseous and liquid phases to soft matters, biological organisms, and even rigid materials.^2^ This pan-media 3D printing strategy opens new frontiers in additive manufacturing, allowing for the fabrication of intricate structures and functional devices via unconventional settings. The following content delves into the LM 3D printing process across various printing media and key post-processing technologies involved with each medium.

Many gases play roles that affect the printing process and fabrication output. In air, oxide layers would form outside LMs, generating semiconducting properties, aiding the adhesion and assembly of structures. Especially for LM composites, where LM particles are separated by polymer film, sintering methods such as laser, chemical processing, or mechanical compression are typically required to restore electrical conductivity (Figure 1Bi). Besides, the reaction of nitrogen-rich gases like ammonia or nitrogen with LMs that include Ga facilitates the production of GaN, which is useful for various semiconductor applications (Figure 1Bi). Additionally, nitrogen gas helps prevent oxidation during the printing process.

For liquid media, it would aid thermal management, enabling rapid cooling and reducing heat accumulation, which improves structural fidelity (Figure 1Bii). Concurrently, the low-oxygen environment of liquid medium helps prevent further oxidation of LMs; moreover, adding specific solvents, such as acids or bases, into solutions can completely remove surface oxides and surfactants to modulate the surface tension of LMs. These features allow for precise management over the printing process, potentially broadening the bio-applications of LM 3D printing in flexible electronics, biomedical devices, and more.

Regarding soft matter media, mechanical strength is a critical factor in overcoming gravitational constraints and directly affects the ability to support precise, complex LM 3D structures. The elasticity of soft matter determines its deformation behavior under the weight of LM structures, which must be carefully balanced to provide adequate support while allowing for the controlled movement of the printing nozzle. Additionally, soft matter media provide an *in situ* encapsulation strategy for 3D-printed LM devices, where mixing acrylamide with nano-clay and using photoinitiation or thermal methods enable rapid curing of the hydrogel matrix for efficient packaging (Figure 1Biii).

Further, organic media-embedded LM 3D printing is an emerging technology in many bio-applications, such as *in vivo* printing and implanted electronics, and poses unique challenges due to the inherent complexity of these environments (Figure 1Biv). Biological tissues typically exhibit nonlinear and anisotropic elastic behavior, which significantly influences the distribution of LMs and stress patterns at the LM-tissue interface. This mechanical complexity impacts both the initial placement and long-term stability of LM structures. To improve the stability and performance, coaxial printing can be employed to encapsulate LM-based devices with silicone rubber.

For rigid media, LM 3D printing presents both challenges and opportunities, primarily through physical adhesion and filling or chemical interfacial reactions. The surface energy of solid substrates, determined by chemical composition and microstructure, significantly influences LM wetting behavior, with surface treatments like plasma processing or PMA coating effectively reducing the surface energy to facilitate LM printing. Notably, interfacial chemical reactions between Ga-based LMs and metals such as Al, Au, and Ag provide a novel approach to LM 3D printing in solid media (Figure 1Bv). The formation of intermetallic compounds at these interfaces enables precise control over surface morphology, opening new avenues for advanced manufacturing techniques. Notably, granular solid materials like soil can also serve as support media for 3D printing, offering a promising pathway for the rapid fabrication of large-scale metallic components (Figure 1Bv).

## Pan-media LM 3D center

From the above description, we can now come up with generalized pan-media printing. By regulating printing inks, media, and fabrication technologies, a series of LM 3D printers based on different printing principles of direct ink writing, field-assisted printing, and multi-material 3D printing can be incorporated to manufacture versatile 3D structures for soft robotics, flexible electronics, and bio-applications. From the first-ever desktop LM printer (Figure 1Ci) to multi-ink 3D printers (Figure 1Cii) up to the latest tension-driven 3D printer (Figure 1Ciii), the optimization of LM printers is crucial for adjusting ink properties, regulating printing platforms, enhancing printing efficiency, and designing printed structures.^4^^,^^5^ Therefore, future LM 3D printers will evolve toward a pan-media center, enabling the delivery of LM and allied inks to target sites across diverse media and, accordingly, directly constructing devices *in situ* to usher in an era where medium boundaries no longer restrict its administration.

Here, we envision a pan-media printing center equipped with functional ink storage, pan-media library, printing head system, and control units designed to meet the targeted printing requirements (Figure 1Di). This system precisely deposits LM inks across various media and holds promise for extending to HMPMs and non-metal 3D printing inks, enabling functional manufacturing beyond conventional methods. Such broad compatibility of inks and media allows printing processing to transcend conventional limitations, facilitating functional manufacturing across a wide spectrum of environments. To facilitate a systematic understanding of the pan-media 3D printing principle as described above and to bridge the envisioned pan-media LM 3D printing center, we also present the relationships between ink materials, media, and printing technologies in a more concise form (Figure 1Dii). The workflow diagram outlines the process from demand input to final product output, covering four key stages of ink storage, media library, media-embedded printing, and post-processing. The platform showcases outstanding adaptability, optimizing key printing technologies to suit the unique properties of different media and the physical characteristics of inks, including factors like melting point, rheology, and electrical conductivity. The selection of different printing inks and media, due to their varying physical and chemical properties, requires different printing approaches and post-processing steps during the fabrication process, ultimately creating customized end-user devices *in situ* as desired. Grounded in the pan-media theory, this platform will significantly expand the possibilities for LM 3D printing, offering a groundbreaking strategy for tackling the complexities of multi-material, multi-functional manufacturing in diverse environments.

## Conclusion

Overall, this article outlines the generalized pan-media principle to unify all 3D printing technologies and materials in one pan-media frame across multi-inks, diverse media, and processing methods, paving the way for building a universal 3D additive manufacturing platform. To a large extent, society would witness the coming of the pan-media fabrication center that breaks the boundaries of inks and media, enabling widespread utilization in intelligent and customized 3D printing scenarios.

## Acknowledgments

This work is partially supported by the 10.13039/100000001Beijing National Science Foundation (L212064) and the 10.13039/501100001809National Natural Science Foundation of China (grant no. 51890893).

## Declaration of interests

The authors declare no competing interests.
